# Effect of Microplastic Contamination on In Vitro Ruminal Fermentation and Feed Degradability

**DOI:** 10.1111/asj.70063

**Published:** 2025-05-27

**Authors:** Khalil Abid, Mohamed Aroua, Salvatore Barbera, Sara Glorio Patrucco, Hatsumi Kaihara, Mokhtar Mahouachi, Samia Ben Saïd, Sonia Tassone

**Affiliations:** ^1^ Department of Agricultural, Forest and Food Sciences University of Turin Grugliasco Turin Italy; ^2^ Laboratoire d'Appui à la Durabilité des Systèmes de Production au Nord‐Ouest Ecole Supérieure d'Agriculture du Kef, University of Jendouba Le Kef Tunisia

**Keywords:** degradability, diet, fermentation, microplastics, rumen

## Abstract

This study examined the effects of microplastic (MP) contamination on rumen fermentation dynamics and concentrate degradability using an in vitro model with lamb rumen fluid. Three types of MPs—polyethylene terephthalate (PET), low‐density polyethylene (LDPE), and polyamide (PA)—were tested at contamination levels of 0%, 0.6%, 1.2%, and 1.8% of dry matter. MP contamination significantly disrupted rumen fermentation dynamics, reduced feed degradability, increased gas production, accelerated fermentation rates, and shortened the lag time before gas production (*p* < 0.05). Additionally, MPs impaired microbial efficiency, increased ammonia‐nitrogen (NH₃‐N) levels, decreased rumen protozoa populations, and reduced concentrate degradability (*p* < 0.05). LDPE exhibited the most severe effects, causing the highest increases in gas production and NH₃‐N levels (15% and 12%, respectively at LDPE highest dose) while decreasing microbial efficiency, protozoa count, and feed degradability (16.0%, 16.4%, and 4.5%, respectively at LDPE highest dose). The severity of MPs' impacts followed a significant linear trend, with higher concentrations leading to more pronounced negative effects. The findings highlight MPs as significant emerging pollutants that can adversely affect rumen function and animal nutrition.

## 1 Introduction

Microplastics (MPs), defined as plastics with a dimension ranging from 1 μm to 5 mm, have emerged as a widespread environmental contaminant with significant implications for livestock systems (Ramachandraiah et al. 2022; Sandil 2024). Recent studies have revealed extensive contamination of ruminant feed by MPs with samples from India and Italy showing 100% contamination across various feed types, involving different MP polymers (Maganti and Akkina 2023; Glorio Patrucco et al. 2024). This contamination is primarily attributed to the extensive use of plastic materials in the feed industry, including plastic packaging for feed ingredients and the use of plastic films for the preservation of silage and nets for hay, which inadvertently introduce MPs into the feed supply chain (Su et al. 2025). Additionally, plants can absorb MPs from contaminated soils through xylem uptake, which leads to their accumulation in edible plant tissues, further exacerbating feed contamination (Li et al. 2023).

Due to their small size, MPs are accidentally ingested by animals during feeding, making feed contamination a route of MP exposure within the animal system (Enyoh et al. 2020; Dong et al. 2023; Khan et al. 2024). A study on sheep proved that most of ingested polystyrene (PS) MPs were excreted via feces within 72 h, with less than 1% translocated into the bloodstream, milk, and urine (Shelver et al. 2024). Moreover, the widespread contamination of ruminant feces with MPs across different continents underscores the extent of this issue: 92% of sheep fecal samples in Spain and 93% of goat fecal samples in Ecuador contained MPs, illustrating the potential for widespread global contamination (Beriot et al. 2021; González‐Puetate et al. 2024).

The toxicological impacts of MPs on ruminants are becoming increasingly evident. In vitro study on goat mammary epithelial cells have demonstrated that PS MPs impaired cell viability, disrupted cellular structures, and induced oxidative stress and apoptosis (Wang et al. 2024). In vivo study on sheep fed with 100 mg of PS MPs per day for 60 days revealed concerning effects, including liver and kidney swelling, alongside reductions in blood parameters, like hemoglobin, thrombocrit, and albumin levels (Chang et al. 2024). Furthermore, studies have reported the detrimental effects of MPs on reproductive health, with PS MPs impairing oocyte maturation in cows and sperm motility in bulls (Grechi et al. 2023; Grechi et al. 2024).

Despite mounting evidence of the negative health impacts of MPs, there is limited research on their effects on the ruminant digestive system. Recently, an in vitro study has indicated that polyethylene terephthalate (PET) MPs in bull ruminal fluid at concentrations of 10 g/L reduced fiber and protein degradability of hay (Tassone et al. 2024). Another in vitro study demonstrated that low‐density polyethylene (LDPE) MPs in lamb ruminal fluid at concentrations of 3.3 g/L reduced organic matter degradability and metabolizable energy from concentrate feed (Tassone et al. 2025). Similarly, an in vivo study on sheep has shown that daily ingestion of 100 mg of PS MPs decreased the apparent digestibility of fiber and fat in diets (Chang et al. 2024). The negative effect of PS MPs is attributed to damage to the jejunal epithelium, reduced rumen papilla length and surface area, and modifications in rumen microbiota abundance (Chang et al. 2024) while the adverse impact of LDPE MPs is linked to a reduction in the rumen protozoa population (Tassone et al. 2025). As a consequence, the ingestion of MPs reduce growth performance, feed conversion efficiency, and meat quality, as observed in sheep ingesting 100 mg of PS MPs daily (Chang et al. 2024).

Given the global prevalence of MP contamination in ruminant feed, it is imperative to explore the impact of different MP polymers on rumen fermentation and feed degradation. This study hypothesizes that the presence of MPs in feed disrupts ruminal fermentation and feed degradability, with these effects being dependent on the type of MP polymer and its concentrations. This study aimed to address these gaps by evaluating the effects of various MP polymers at different concentrations on rumen fermentation kinetics and feed degradability.

## 2 Ethical Approval

The rumen fluid sampling protocol was approved by the Animal Welfare and Use Committee of the Ethics Committee ‐ National School of Veterinary Medicine, IACUC, ENMV‐Sidi Thabet, Tunisia (CEEA number ‐ ENMV 35/21: 2021).

## 3 Materials and Methods

### 3.1 Concentrate and Microplastic Samples

The feed employed in the experiment was the same concentrate used in the diet of the rumen fluid donor lambs. The concentrate was composed of 800 g of barley, 175 g of soybean meal, and 25 g of mineral and vitamin supplement per kg of dry matter (DM). It was ground into powder using a Retsch mill (Retsch ZM200, Retsch GmbH, Haan, Germany), sieved (mesh size 1 mm), and stored in a dark glass bottle in a dry and cool place until analysis to avoid further uncontrolled environmental MP contamination.

DM (AOAC 930.15), crude protein content (AOAC 954.01), and ash content (AOAC 942.05) were determined according to the methods of the Association of Official Analytical Chemists (2000). Neutral detergent fiber (NDF), acid detergent fiber (ADF), and acid detergent lignin were determined according to the methods proposed by Van Soest et al. (1991), using an ANKOM220 fiber analyzer (ANKOM Technology, Macedon, New York, United States).

The ground concentrate was used pure and polluted with three types of MPs: PET, LDPE, and polyamide (PA) at three different doses (0.6%, 1.2%, and 1.8% on a dry weight base of concentrate). The lowest dose was used based on the study conducted by Galyon et al. (2023) on Holstein bull calves fed 2.3 kg of concentrate supplemented with 13.65 g of LDPE macroplastic. The concentrate without MP addition was regarded as a control similar to the study conducted by Galyon et al. (2023).

**FIGURE 1:**
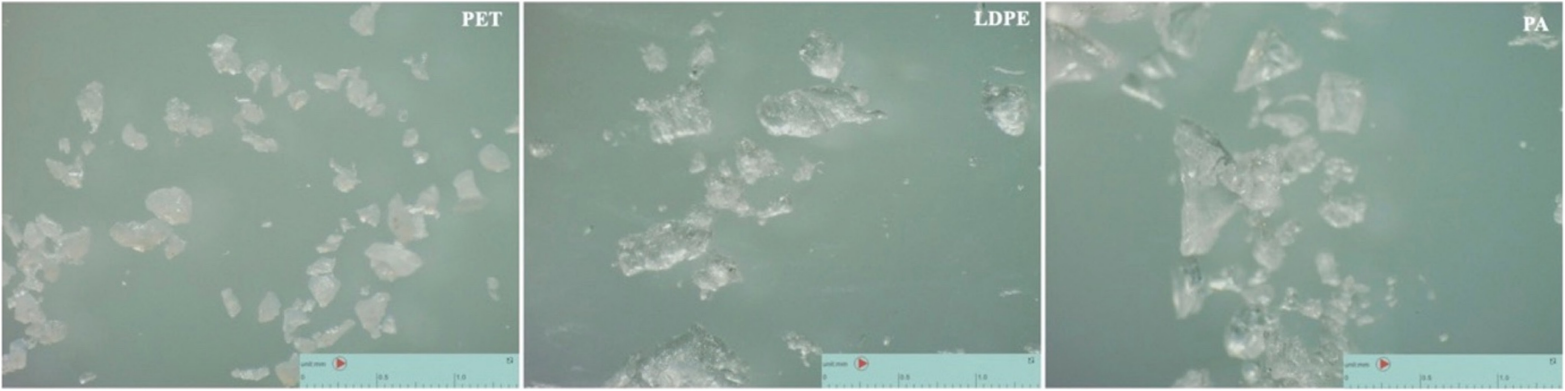
Stereomicroscope pictures of the microplastics used in the experiment.

**FIGURE 2:**
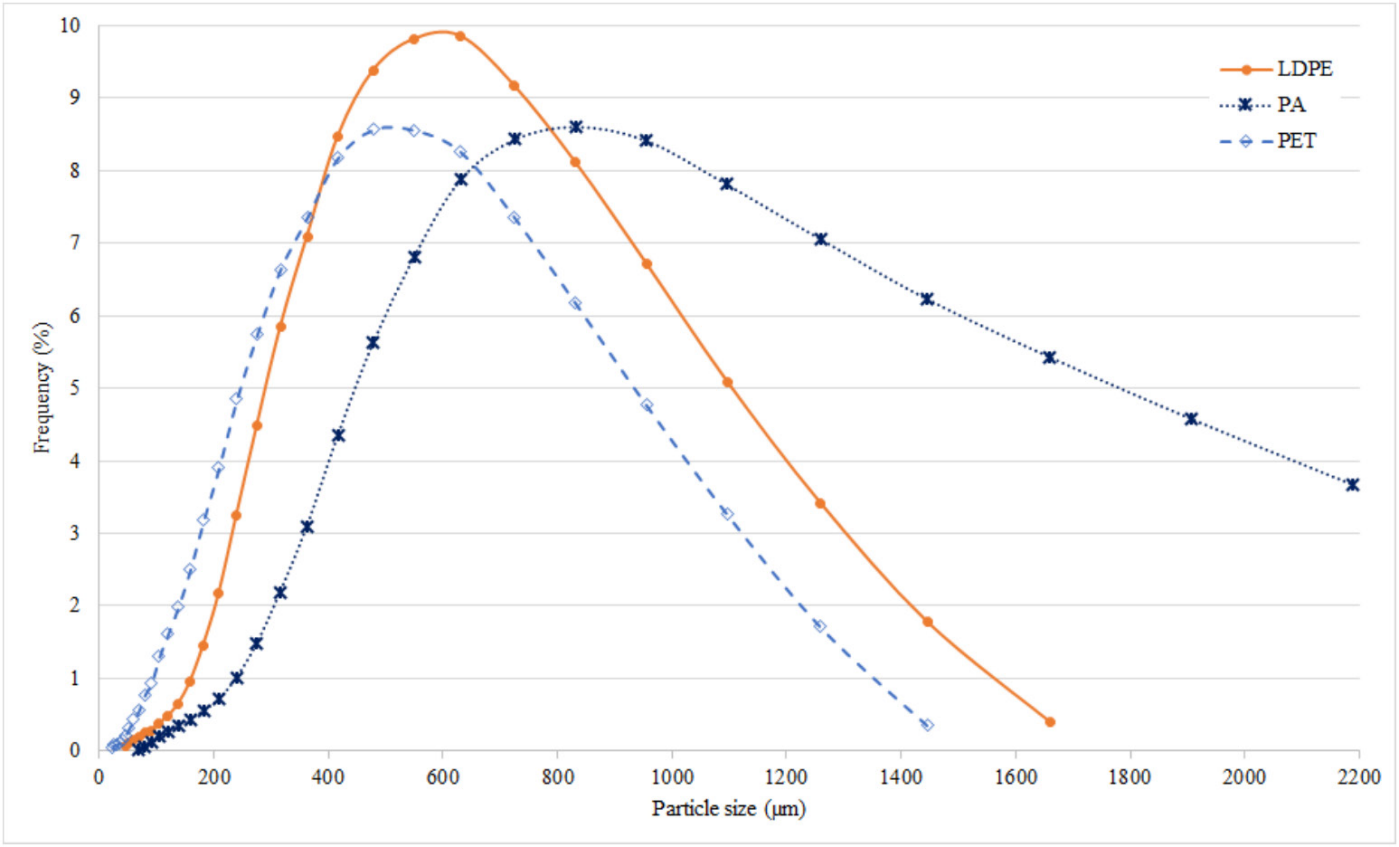
Particle size distribution of each microplastics polymer used in the experiment based on particle counts.

The pictures of the MP powders were taken with a stereomicroscope (Nikon H550S, Japan), as shown in Figure 1. MP powders were provided by an Italian company with the following characteristics: PET with an average density of 1.38 g/cm^3^ and average size of 522 μm; LDPE with an average density of 0.918 g/cm^3^ and average size of 643 μm; and PA with an average density of 1.14 g/cm^3^ and average size of 993 μm. The distribution of the dimensions of the three types of MPs is illustrated in Figure 2 based on data provided by the company.

### 3.2 In Vitro Rumen Fermentation

Rumen fluid was collected from five male lambs of Queue Fine de l'Ouest, aged 9 months and weighing 30 kg. These lambs were fed 0.6 kg of oat hay and 0.3 kg of concentrate, the chemical composition of which is shown in Table 1 and had free access to fresh water. Rumen fluid was collected before the morning meal using a rubber stomach tube inserted into the rumen through the esophagus and a hand pump according to the protocol described by Muizelaar et al. (2020). The rumen fluid sampling protocol was approved by the Animal Welfare and Use Committee of the Ethics Committee ‐ National School of Veterinary Medicine, IACUC, ENMV‐Sidi Thabet, Tunisia (CEEA number ‐ ENMV 35/21: 2021). Rumen fluid samples were immediately filtered through four layers of cheesecloth at 39 °C under continuous flushing with carbon dioxide (CO_2_). The filtered rumen fluid was mixed in a 1:2 ratio (V:V) with buffer solution under constant rinsing with CO_2_. The buffer solution was freshly prepared according to the method described by Menke and Steingass (1988). It consisted of macromineral, micromineral, resazurin solution, and distilled water.

**TABLE 1:** Chemical composition of lamb diet (g/kg dry matter).

Item	Concentrate	Oat hay
Dry matter^a^	911	904
Ash	41	80
Crude protein	161	80
Neutral detergent fiber	131	530
Acid detergent fiber	32	350
Acid detergent lignin	8	77

^a^ Dry matter on g/kg fresh matter.

Two hundred milligrams of control concentrate samples and concentrate with the appropriate dose and type of MPs were weighed in triplicate into black glass serum bottles of 120 mL capacity. This process was carried out with utmost precision to ensure the accurate incorporation of the desired level of MP contamination in each serum bottle. Each bottle was then inoculated with 30 mL of buffered rumen fluid (20 mL of buffer and 10 mL of rumen fluid), resulting in a total of 30 bottles. In addition, three serum bottles with only buffered rumen fluid were used as blanks to determine gas production from the buffered rumen *inoculum*. Additionally, three serum bottles containing 200 mg of LDPE with buffered rumen fluid, three serum bottles containing 200 mg of PET with buffered rumen fluid, and three serum bottles containing 200 mg of PA with buffered rumen fluid were used to determine gas production from the MPs alone.

All bottles were immediately sealed with a rubber cap and an aluminum crimp cap and incubated in a shaking water bath at 39 °C and 120 rpm for 96 h. The gas pressure in the headspace of each fermentation bottle was measured at 2, 4, 6, 8, 12, 24, 48, 72, and 96 h after incubation by inserting a 23‐gauge needle attached to a pressure transducer (model PX4200‐015GI, Omega Engineering Inc., Laval, QC, Canada) into the rubber stoppers of the serum bottles. The needles were left on the serum bottles after the insertion to allow the release of all available gas from the glass serum bottles.

The recorded gas pressure was converted to the volume of gas produced at each measuring time, using the equation described by Jabri et al. (2022):

$$\begin{array}{c} \mathrm{Gas}\;\text{volume}\;\text{produced}=\mathrm{Gas}\;\text{pressure}\;\text{recorded}\;\times \frac{\mathrm{Vf}-\mathrm{Vi}}{\text{Patm}} \end{array}$$

where gas volume produced is expressed in mL/g DM; gas pressure is recorded in bar; *Vf* is the volume of the incubation bottle (mL); *Vi* is the volume of *inoculum* added to each bottle (mL); and *P*_atm_ is the atmospheric pressure (bar).

Three different incubation runs were performed in three consecutive weeks.

### 3.3 Rumen Activity Evaluation

**FIGURE 3:**
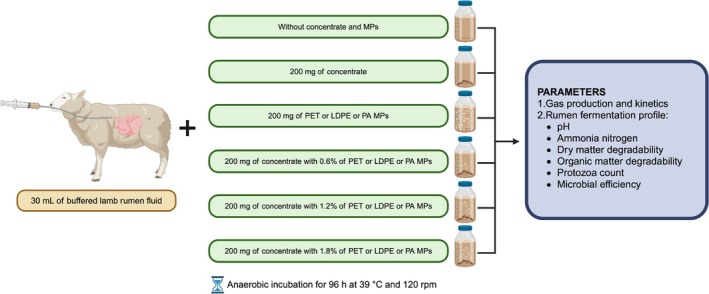
Summary of the experimental design.

The influence of MPs on rumen activity was evaluated by measuring: gas production and kinetics and rumen fermentation profile, as detailed. The experimental design is summarized in Figure 3.

#### 3.3.1 Gas Production and Kinetics

The asymptotic gas production (B), the gas production rate (C), and the onset time of gas production (Lag) were calculated by fitting the data of the cumulative volume of biogas production (GP) with an exponential mathematical model according to France et al. (2000):

$${\mathrm{GP}}_{\left(t\right)}=B\times \left(1-e^{-C\;\left(t-\mathrm{Lag}\right)}\right)$$

where GP_(*t*)_ is the net gas production (mL/g DM) on the time; *t* is the incubation time (h); *B* is the asymptotic gas production (mL/g DM); C is constant gas production rate (/h); and Lag is onset time of gas production (h).

The time to half‐maximal gas production was calculated as:

$$T_{1/2}=\frac{\mathrm{Ln}\;2}{C}+\mathrm{lag}$$

where T_1/2_ is the time to half‐maximal gas production (hours); C is constant gas production rate (/hour); and Lag is onset time of gas production (hours).

The average fermentation rate (AFR) was defined as the average gas production rate between the start of the incubation and *T*_1/2_ and was calculated as follows:

$$\mathrm{AFR}=\frac{B\times C}{2\;\left[\ln\;2+C\times \mathrm{Lag}\right]}$$

where AFR is the average fermentation rate (mL/h); *B* is the asymptotic gas production (mL/g DM); C is the constant gas production rate (/h); and Lag is the onset time of gas production (h).

#### 3.3.2 Rumen Fermentation Profile

##### 3.3.2.1 pH

The rumen pH from each bottle was immediately measured at the end of fermentation using a pH meter (Thermo Scientific Orion Star A221, pH Portable Meter).

##### 3.3.2.2 Ammonia‐Nitrogen

The rumen ammonia‐nitrogen (NH_3_‐N) from each bottle was determined at the end of fermentation. Briefly, 5 mL of supernatant from each bottle was collected and preserved by adding 2 mL of 1 N H_2_SO_4_. NH_3_‐N was measured using the micro‐Kjeldahl method as described by the Association of Official Analytical Chemists (2000).

##### 3.3.2.3 Dry Matter Degradability and Organic Matter Degradability

The DM degradability (DMD) and the organic matter degradability (OMD) of control concentrate and concentrate with MP addition were determined at the end of fermentation. Briefly, the content of each serum bottle was filtered using filter paper disks (Whatman 541; Whatman Scientific Ltd, Maidstone, Kent, England), and the incubation residues were dried overnight at 105 °C to determine the DMD using the formula:

$$\mathrm{DMD}\;\left(\%\right)=\frac{\text{initial}\;\mathrm{dry}\;\text{matter}-\text{residual}\;\mathrm{dry}\;\text{matter}\;}{\text{initial}\;\mathrm{dry}\;\text{matter}}\times 100$$

Following this, the incubation residues were incinerated at 550 °C to determine the OMD using the formula:

$$\mathrm{OMD}\;\left(\%\right)=\frac{\text{initial organic matter}-\text{residual organic matter}\;}{\text{initial organic matter}}\times 100$$

##### 3.3.2.4 Protozoa Count

The concentration of rumen protozoa in the rumen fluid was determined at the end of rumen fermentation according to the protocol of Dehority (1993). Briefly, 0.5 mL of supernatant was collected from each bottle and mixed with 2 mL of physiological methyl green formalin solution and shaken gently. The mixture was pipetted into a Levy–Hausser counting chamber (Husser Scientific, Horsham, Pennsylvania, United States) and the number of rumen protozoa was counted under light microscopy.

##### 3.3.2.5 Microbial Efficiency

Microbial efficiency was determined through the partitioning factor (PF) that was calculated as the ratio between the degraded DM of the feed after 96 h of incubation (mg/g DM) to the total gas volume produced from the feed over the same period (mL/g DM), according to Blümmel et al. (1997).

### 3.4 Statistical Analyses

All collected data were analyzed as an augmented 3 × 3 factorial plus one control design. The statistical model included the following factors: control (concentrate not contaminated), MP type (LDPE, PET, and PA), MP dose (0.6%, 1.2%, and 1.8% in the concentrate), and the interaction between MP type and MP dose in the concentrate. The analysis was performed using the PROC MIXED of SAS version 9.1 (SAS Institute, Cary, North Carolina, United States). For each type of MPs, the linear and quadratic effects of increasing the dose were evaluated using the polynomial contrast statement of SAS Institute Inc. Additionally, Tukey's multiple range test was applied to compare differences between treatment means. The differences were considered significant when *p* < 0.05.

## 4 Results and Discussion

This study revealed significant effects of various MP polymers and concentrations present in lamb feed. Specifically, it highlighted alterations in rumen gas production kinetics, fermentation profiles, and feed degradability.

### 4.1 Gas Production and Kinetics

**FIGURE 4:**
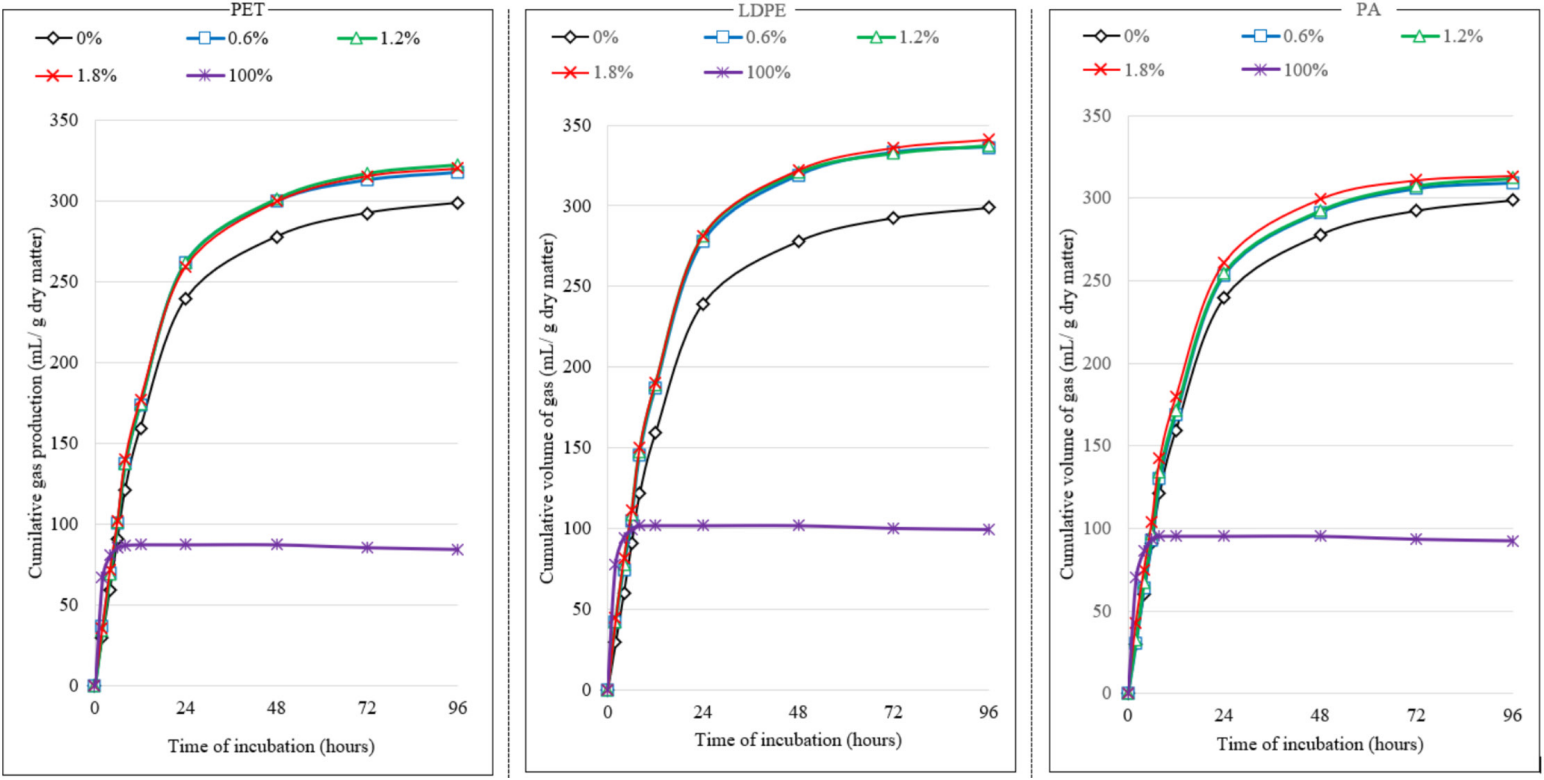
The impact of microplastic type and dose on gas production during in vitro rumen concentrate fermentation.

The contamination of concentrate with various MP polymers significantly increased gas production during ruminal fermentation, as illustrated in Table 2 and Figure 4. This increase in gas emissions, particularly at the early stages of fermentation, can be attributed to high gas emission from MPs (Figure 4). This gas production resulted from the hydrolysis of MPs by esterases, lipases, proteases, and cutinases, enzymes produced by rumen bacteria, eukaryotes, and archaea. These enzymes lead to the release of terephthalic acid, mono‐2‐hydroxyethyl terephthalate, and bis(2‐hydroxyethyl) terephthalate, as demonstrated in the degradation of PET MPs (Quartinello et al. 2021) as well as degradation of portions of LDPE and PET MPs (Kaihara et al. 2024). However, to the best of our knowledge, no information is currently available regarding the intermediate degradation products of LDPE and PA in the ruminal environment, nor the specific gas production profiles associated with the ruminal degradation of MPs. In soil under anaerobic conditions, previous studies have shown that microbial degradation of various MP polymers leads to the production of methane and CO₂ (Li et al. 2024) and recent meta‐analysis demonstrated that soil contamination with different MP polymers also leads to increased gas emissions (Iqbal et al. 2024). Despite the differences conditions between the soil and rumen, these findings point to the possibility that the effect of MPs may follow similar mechanisms in both environments.

**TABLE 2:** The impact of microplastic type and dose on gas production kinetics in vitro lamb rumen.

Types	MP doses (%)	Gas production kinetics				
		B (mL/g DM)	C (/h)	Lag (h)	T_1/2_ (h)	AFR (mL/h)
CC	0.0	292.5d	0.0702bc	0.77a	10.65a	13.83d
CC + PET	0.6	306.7c	0.0734ab	0.55ab	10.02abc	15.43c
	1.2	311.9bc	0.0719abc	0.38bc	10.04abc	15.61ab
	1.8	309.7bc	0.0711bc	0.26bc	10.06abc	15.51c
CC + LDPE	0.6	328.9ab	0.0697bc	0.38bc	10.37ab	15.99abc
	1.2	322.9ab	0.0738ab	0.23bc	9.63cd	16.80a
	1.8	335.2a	0.0687c	0.35bc	10.51ab	16.20abc
CC + PA	0.6	300.9dc	0.0736ab	0.39bc	9.82bcd	15.34c
	1.2	304.5dc	0.0710bc	0.29bc	10.10abc	15.18c
	1.8	307.7c	0.0753a	0.09d	9.31d	16.56ab
CC + PET	Linear	0.672	0.756	0.004	0.903	0.920
	Quadratic	0.556	0.676	0.734	0.995	0.841
CC + LDPE	Linear	0.518	0.651	0.759	0.735	0.830
	Quadratic	0.277	0.026	0.153	0.035	0.398
CC + PA	Linear	0.365	0.349	0.002	0.440	0.046
	Quadratic	0.976	0.053	0.563	0.017	0.167
SEM		8.60	0.0029	0.247	0.424	0.701
*p* value	CC vs CC + MPs^a^	0.002	0.257	< 0.001	0.013	0.001
	Type	< 0.001	0.110	0.041	0.093	0.192
	Dose	0.472	0.876	< 0.001	0.745	0.562
	Type × dose	0.748	0.026	0.116	0.183	0.489

Values within column with no common letters (a–d) differ significantly (*p* value < 0.05).

Abbreviations: AFR, average fermentation rate; B, asymptotic gas production; C, constant gas production rate; CC, concentrate; DM, dry matter; Lag, onset time of rumen gas production; LDPE, low‐density polyethylene; MPs, microplastics; PA, polyamide; PET, polyethylene terephthalate; SEM, standard error of the mean; T_1/2_, time to half‐maximal gas production.

^a^ Contrasts comparing control concentrate vs. all concentrates contaminated with MPs.

The current study further demonstrated that the impact of MPs on the gas formation process in the rumen is closely linked to the specific type of MPs. Concentrates contaminated with LDPE produced more gas compared to those with PA or PET (Figure 4). At the highest dose (1.8%), gas production from LDPE‐contaminated concentrate was 15% higher than the control, while the increases for PET and PA were 6% and 5%, respectively (Table 2). The differential effect of various MP polymers on gas formation can be attributed to their distinct chemical composition and physical structure. To the best of our knowledge, no previous studies have evaluated the effect of different types of MPs on ruminal gas emissions. In contrast, studies conducted in other ecosystems, particularly in soil, have demonstrated that the impact of MPs on gas emissions is highly dependent on the polymer type (Iqbal et al. 2024). However, increasing the dose of MPs did not further influence gas formation in the rumen.

The presence of MPs also significantly increased the AFR. At the highest MP concentration, the AFR increased by 20% for PA, 18% for LDPE, and 13% for PET compared to the control (Table 2). However, neither the type nor the dose of MPs appeared to significantly affect the AFR, indicating that while MPs accelerated fermentation, this effect might not scale with polymer concentration.

This acceleration in the AFR was accompanied by a substantial reduction in the Lag time before gas production. At the highest MP dose, the Lag time decreased from 0.77 h in the control to 0.35, 0.26, and 0.09 h for the concentrates contaminated with LDPE, PET, and PA, respectively (Table 2). The reduction in the Lag time was more pronounced with higher concentrations of PET and PA, indicating a linear relationship between these MPs and the acceleration of fermentation onset. This effect can be explained by MPs may act as nucleation sites for gas bubble formation, further promoting early gas production during fermentation (Nuelle et al. 2014). Moreover, MPs may attract decomposing bacteria, indirectly promoting gas formation as proved in soil study (Bian et al. 2022).

### 4.2 Rumen Fermentation Profile

Results of rumen fermentation profile are presented in Table 3.

**TABLE 3:** The impact of microplastic type and dose on rumen fermentation profile in vitro lamb rumen.

Type	MP doses (%)	Rumen fermentation profile					
		pH	NH_3_‐N (mg/100 mL)	OMD (mg/g)	DMD (mg/g)	Protozoa (10^5^/mL)	PF
CC	0.0	6.54	24.90d	721.2a	671.9a	4.38a	2.272a
CC + PET	0.6	6.56	25.51bcd	712.1ab	666.8ab	4.18abc	2.164b
	1.2	6.56	25.70bc	697.7bc	652.7bc	4.24ab	2.077cde
	1.8	6.53	26.56bc	688.3c	644.4c	3.87cd	2.057de
CC + LDPE	0.6	6.55	25.60bcd	717.4a	672.6a	4.16abc	2.044e
	1.2	6.54	26.84ab	699.4bc	655.9bc	3.92bcd	2.032e
	1.8	6.53	27.91a	685.1c	641.8c	3.66d	1.908f
CC + PA	0.6	6.54	25.01cd	705.0ab	661.4ab	4.23ab	2.182b
	1.2	6.53	25.29bcd	699.2bc	655.9bc	4.20abc	2.135bc
	1.8	6.52	26.32bc	696.1bc	652.1bc	3.81d	2.117bcd
CC + PET	Linear	0.322	0.092	0.014	0.022	0.002	0.006
	Quadratic	0.847	0.770	0.645	0.713	0.551	0.291
CC + LDPE	Linear	0.452	0.044	0.005	0.006	< 0.0001	0.001
	Quadratic	0.876	0.880	0.886	0.885	0.651	0.079
CC + PA	Linear	0.432	0.050	0.251	0.251	0.001	0.041
	Quadratic	0.782	0.760	0.898	0.898	0.743	0.585
SEM		0.034	1.035	9.98	10.64	0.240	0.0461
*p* value	CC vs CC + MPs^a^	0.771	< 0.001	0.046	0.028	< 0.0001	< 0.001
	Type	0.851	0.191	0.942	0.709	0.04	< 0.001
	Dose	0.445	0.047	< 0.0001	< 0.001	< 0.001	< 0.001
	Type × dose	0.667	0.331	0.564	0.587	0.501	0.129

Values within column and type with no common letters (a–f) differ significantly (*p* value < 0.05).

Abbreviations: CC, concentrate; DMD, dry matter degradability; LDPE, low‐density polyethylene; MPs, microplastics; NH_3_‐N, rumen ammonia‐nitrogen on mg/100 mL; OMD, organic matter degradability; PA, polyamide; PET, polyethylene terephthalate; PF, partition factor on mg degraded dry matter of the feed after 96 h of incubation/mL gas produced from the feed after 96 h of incubation; pH, rumen pH; SEM, standard error of the mean.

^a^ Contrasts comparing control concentrate vs. all concentrates contaminated with MPs.

#### 4.2.1 pH

Throughout the experiment, the rumen pH remained steady at an average of 6.5. Despite the influence of MPs on rumen fermentation and concentrate degradability, there was no discernible impact on the rumen pH. A similar trend was found in an in vivo study on lambs ingested PS MPs at 100 mg/day (Chang et al. 2024). These results from different MP polymers reinforce the idea that MPs do not appear to modify rumen pH.

#### 4.2.2 Ammonia‐Nitrogen Concentration

Quantification of NH_3_‐N concentration holds significant importance as it serves as a key indicator of nitrogen utilization efficiency (Benetel et al. 2022). In this context, our study showed that concentrate contaminated with MPs led to an increase in concentration of NH_3_‐N during rumen fermentation (Table 3). However, no significant differences were observed between the types of MPs. The effect on NH_3_‐N concentration was related to the level of MPs, showing a significant linear increase with higher levels of LDPE and a similar trend for PET and PA. At the highest dose of contamination (1.8%) by LDPE, PET, and PA, the NH_3_‐N in the rumen environment increased by 12.1%, 6.7%, and 5.7%, respectively, compared to control concentrate.

This disruption of the nitrogen utilization pathway negatively impacts animal nutrition by increasing nitrogen wastage as well as decreasing ruminant performance and thus impairing environmental sustainability through ecological problems (Al‐Marzooqi et al. 2021).

The presence of MPs led to a significant reduction in rumen protozoa with a linear relationship observed as MP levels increased, particularly pronounced for LDPE (Table 3). Although such reductions are typically associated with lower NH₃‐N concentrations (Spanghero et al. 2022), this was not observed in our study where NH₃‐N increased. This discrepancy suggests that the impact of MPs may selectively reduce certain rumen protozoa species responsible for decreasing NH_3_‐N levels such as *Isotricha* spp. and holotrich *Dasytricha* that have a high capacity to reduce ruminal NH_3_‐N levels (Jouany 1996). Similarly, studies conducted in other ecosystems, particularly in compost, have demonstrated that the presence of polyethylene MPs at a concentration of 0.5% in compost increased NH_3_‐N emissions (Sun et al. 2020). However, Chang et al. (2024) found that ingestion of PS MPs lowered lamb rumen NH₃‐N concentrations, highlighting the polymer‐specific nature of these effects.

#### 4.2.3 Dry Matter Degradability, Organic Matter Degradability, and Protozoa

In contrast to gas production, the presence of MPs in the concentrate feed decreased both DMD and OMD of the concentrate. A similar inverse relationship between gas production and feed degradation was noted with the inclusion of essential oils from marjoram and basil as rumen additives (Selim et al. 2021). The negative impact of MPs on degradability aligns with findings by Chang et al. (2024) who demonstrated that the ingestion of PS MPs lowered the apparent dry matter digestibility of lambs' diets. In our study, the increase in the levels of LDPE and PET MPs in the concentrate caused a linear decrease in both DMD and OMD. Specifically, DMD dropped by 4.5%, 4.1%, and 3.0%, and OMD by 5.0%, 4.6%, and 3.5% for the concentrates contaminated with high level of LDPE, PET, and PA, respectively, compared to the control (Table 3). However, the type of MP polymer did not have a significant effect on either DMD or OMD. This decline in ruminal degradability could be attributed to several factors, including the low ruminal degradation capacity to degrade MPs, with less than 1% degradation observed for LDPE and PET MPs in buffered rumen fluid (Kaihara et al. 2024), the release of toxic compounds from MPs in the rumen environment (Liao and Yang 2022), the alteration of ruminal microbiota composition (Chang et al. 2024), and a reduction of protozoa in the rumen as noted in Table 3 . Similarly, a previous study conducted in other ecosystems by Zhang et al. (2021) demonstrated that MPs reduced the growth, abundance, body size, and biomass of marine protozoa in marine ecosystems, which may suggest that a similar mechanism could be at play in the rumen environment, further impairing digestibility.

#### 4.2.4 Microbial Efficiency

The PF, defined as the ratio of dry decomposition to total gas production, serves as a valuable indicator of microbial efficiency (Blümmel et al. 1997). In the present study, it was evident that the inclusion of MPs in concentrate lowered microbial efficiency. Notably, LDPE had more pronounced adverse effects, resulting in a 16.0% reduction in PF at the highest contamination dose (1.8%). Furthermore, the negative impact of MPs on microbial efficiency was intensified in a dose‐dependent manner, following a linear trend across all types of MPs tested. This reduction in microbial efficiency hinders animals to effectively extract nutrients from the feed, ultimately decreasing animal productivity and profitability for ruminant producers. Additionally, diminished microbial efficiency can lead to digestive disorders and metabolic issues in ruminants such as incomplete feed fermentation in the rumen. This inefficiency not only increases gas emissions per unit of degraded feed but also results in greater nutrient loss through faces, contributing to environmental pollution (Blümmel et al. 2003). Similarly, a previous study conducted in other ecosystems by Sun et al. (2020) reported that the presence of polyethylene MPs at a concentration of 0.5% negatively impacted compost quality by reduced organic matter degradability and elevating gas emissions. These parallel results highlight the broader ecological risks associated with MPs.

Lamb rumen activity is significantly impaired by the presence of microplastics (LDPE, PET, and PA) in concentrate feed at concentrations of 0.6%, 1.2%, and 1.8%. Future research should prioritize developing effective strategies to reduce microplastic contamination in animal feeds, with special emphasis on limiting LDPE, which is widely used throughout agricultural operations.

## Conflicts of Interest

The authors declare no conflicts of interest.

## References

[asj70063-bib-0001] Al‐Marzooqi, W. , S. M. Sallam , O. Alqaisi , and H. M. El‐Zaiat . 2021. “Potential of Graded Doses of Neem (*Azadirachta indica*) Seed Oil on Ruminal Fermentation Characteristics, Degradability, and Methane Formation in vitro.” Annals of Animal Science 22, no. 3: 993–999. 10.2478/aoas-2021-0073.

[asj70063-bib-0002] Association of Official Analytical Chemists . 2000. Official Methods of Analysis. 7th ed. Association of Official Analytical Chemists.

[asj70063-bib-0003] Benetel, G. , T. D. S. Silva , G. M. Fagundes , et al. 2022. “Essential Oils as *in Vitro* Ruminal Fermentation Manipulators to Mitigate Methane Emission by Beef Cattle Grazing Tropical Grasses.” Molecules 27, no. 7: 2227. 10.3390/molecules27072227. 35408626 PMC9000866

[asj70063-bib-0004] Beriot, N. , J. Peek , R. Zornoza , V. Geissen , and E. H. Lwanga . 2021. “Low Density‐Microplastics Detected in Sheep Faeces and Soil: A Case Study From the Intensive Vegetable Farming in Southeast Spain.” Science of the Total Environment 755: 142653. 10.1016/j.scitotenv.2020.142653. 33069476

[asj70063-bib-0005] Bian, W. , L. An , S. Zhang , et al. 2022. “The Long‐Term Effects of Microplastics on Soil Organomineral Complexes and Bacterial Communities From Controlled‐Release Fertilizer Residual Coating.” Journal of Environmental Management 304: 114193. 10.1016/j.jenvman.2021.114193. 34864411

[asj70063-bib-0006] Blümmel, M. , A. Karsli , and J. R. Russell . 2003. “Influence of Diet on Growth Yields of Rumen Micro‐Organisms *In Vitro* and *In Vivo*: Influence on Growth Yield of Variable Carbon Fluxes to Fermentation Products.” British Journal of Nutrition 90, no. 3: 625–634. 10.1079/BJN2003934. 13129469

[asj70063-bib-0007] Blümmel, M. , H. P. S. Makkar , and K. Becker . 1997. “ *In Vitro* Gas Production: A Technique Revisited.” Journal of Animal Physiology and Animal Nutrition 77, no. 1–5: 24–34. 10.1111/j.1439-0396.1997.tb00734.x.

[asj70063-bib-0008] Chang, X. , Y. Li , Y. Han , et al. 2024. “Polystyrene Exposure Induces Lamb Gastrointestinal Injury, Digestive Disorders and Inflammation, Decreasing Daily Gain, and Meat Quality.” Ecotoxicology and Environmental Safety 277: 116389. 10.1016/j.ecoenv.2024.116389. 38657458

[asj70063-bib-0009] Dehority, B. A. 1993. Laboratory Manual for Classification and Morphology of Rumen Ciliate Protozoa. CRC Press.

[asj70063-bib-0010] Dong, X. , X. Liu , Q. Hou , and Z. Wang . 2023. “From Natural Environment to Animal Tissues: A Review of Microplastics (Nanoplastics) Translocation and Hazards Studies.” Science of the Total Environment 855: 158686. 10.1016/j.scitotenv.2022.158686. 36099943

[asj70063-bib-0011] Enyoh, C. E. , L. Shafea , A. W. Verla , et al. 2020. “Microplastics Exposure Routes and Toxicity Studies to Ecosystems: An Overview.” Environmental Analysis, Health and Toxicology 35, no. 1: e2020004. 10.5620/eaht.e2020004. 32570999 PMC7308665

[asj70063-bib-0012] France, J. , J. Dijkstra , M. S. Dhanoa , S. Lopez , and A. Bannink . 2000. “Estimating the Extent of Degradation of Ruminant Feeds From a Description of Their gas Production Profiles Observed *In Vitro*: Derivation of Models and Other Mathematical Considerations.” British Journal of Nutrition 83, no. 2: 143–150. 10.1017/S0007114500000180. 10743493

[asj70063-bib-0013] Galyon, H. , S. Vibostok , J. Duncan , et al. 2023. “Clearance of Biodegradable Polymer and Polyethylene Films From the Rumens of Holstein Bull Calves.” Animals 13, no. 5: 928. 10.3390/ani13050928. 36899785 PMC10000221

[asj70063-bib-0014] Glorio Patrucco, S. , L. Rivoira , M. C. Bruzzoniti , S. Barbera , and S. Tassone . 2024. “Development and Application of a Novel Extraction Protocol for the Monitoring of Microplastic Contamination in Widely Consumed Ruminant Feeds.” Science of the Total Environment 947: 174493. 10.1016/j.scitotenv.2024.174493. 38969126

[asj70063-bib-0015] González‐Puetate, I. , G. Martínez‐Cepeda , P. Torres‐Lasso , K. Chávez , and G. Guevara . 2024. “Microplastics in Ruminant Feces in Ecuador.” Ciencia Veterinaria 26, no. 2: 114–129. 10.19137/cienvet202426203.

[asj70063-bib-0016] Grechi, N. , S. Devkota , G. Ferronato , and M. Ferraz . 2024. “171 Microplastics Are Present in Bull Epididymal Sperm and Polystyrene Bead Affects Bovine Sperm Inducing Oxidative Stress on Embryos.” Reproduction, Fertility and Development 36, no. 2: 239. 10.1071/RDv36n2Ab171.

[asj70063-bib-0017] Grechi, N. , R. Franko , R. Rajaraman , et al. 2023. “Microplastics Are Present in Women's and Cows' Follicular Fluid and Polystyrene Microplastics Compromise Bovine Oocyte Function *in Vitro* .” bioRxiv, 2022, 11.04.514813. 10.1101/2022.11.04.514813.

[asj70063-bib-0018] Iqbal, S. , J. Xu , M. S. Arif , et al. 2024. “Could Soil Microplastic Pollution Exacerbate Climate Change? A Meta‐Analysis of Greenhouse Gas Emissions and Global Warming Potential.” Environmental Research 252: 118945. 10.1016/j.envres.2024.118945. 38631466

[asj70063-bib-0019] Jabri, J. , H. Ammar , K. Abid , et al. 2022. “Effect of Exogenous Fibrolytic Enzymes Supplementation or Functional Feed Additives on *in Vitro* Ruminal Fermentation of Chemically Pre‐Treated Sunflower Heads.” Agriculture 12, no. 5: 696. 10.3390/agriculture12050696.

[asj70063-bib-0020] Jouany, J. P. 1996. “Effect of Rumen protozoa on Nitrogen Utilization by Ruminants.” Journal of Nutrition 126: 1335S–1346S. 10.1093/jn/126.suppl_4.1335S. 8642481

[asj70063-bib-0021] Kaihara, H. , K. Abid , S. Barbera , S. Glorio Patrucco , and S. Tassone . 2024. “Assessment of ANKOM DaisyII Incubator for Measuring Microplastic Degradability in the Rumen Environment.” In 2024 International Workshop on Measurements and Applications in Veterinary and Animal Sciences (pp. 1–1). IEEE MeAVeAS. April 22–24, Turin, Italy.

[asj70063-bib-0022] Khan, A. , A. Qadeer , A. Wajid , et al. 2024. “Microplastics in Animal Nutrition: Occurrence, Spread, and Hazard in Animals.” Journal of Agriculture and Food Research 17: 101258. 10.1016/j.jafr.2024.101258.

[asj70063-bib-0023] Li, H. , X. Chang , J. Zhang , et al. 2023. “Uptake and Distribution of Microplastics of Different Particle Sizes in Maize (*Zea mays*) Seedling Roots.” Chemosphere 313: 137491. 10.1016/j.chemosphere.2022.137491. 36493893

[asj70063-bib-0024] Li, K. , L. Du , C. Qin , N. Bolan , H. Wang , and H. Wang . 2024. “Microplastic Pollution as an Environmental Risk Exacerbating the Greenhouse Effect and Climate Change: A Review.” Carbon Research 3, no. 1: 9. 10.1007/s44246-023-00097-7.

[asj70063-bib-0025] Liao, Y. L. , and J. Y. Yang . 2022. “The Release Process of cd on Microplastics in a Ruminant Digestion *in Vitro* Method.” Process Safety and Environmental Protection 157: 266–272. 10.1016/j.psep.2021.11.026.

[asj70063-bib-0026] Maganti, S. S. , and R. C. Akkina . 2023. “Detection and Characterisation of Microplastics in Animal Feed.” Online Journal of Animal and Feed Research 13, no. 5: 348–356. 10.51227/ojafr.2023.50.

[asj70063-bib-0027] Menke, K. H. , and H. Steingass . 1988. “Estimation of the Energetic Feed Value Obtained From Chemical Analysis and in Vitro Gas Production Using Rumen Fluid.” Animal Research and Development 28: 7–55.

[asj70063-bib-0028] Muizelaar, W. , P. Bani , B. Kuhla , et al. 2020. “Rumen Fluid Sampling via Oral Stomach Tubing Method.” In Mesgaran SD, Baumont R., Munksgaard L., Humphries.

[asj70063-bib-0029] Nuelle, M. T. , J. H. Dekiff , D. Remy , and E. Fries . 2014. “A New Analytical Approach for Monitoring Microplastics in Marine Sediments.” Environmental Pollution 184: 161–169. 10.1016/j.envpol.2013.07.027. 24051349

[asj70063-bib-0030] Quartinello, F. , K. Kremser , H. Schoen , et al. 2021. “Together Is Better: The Rumen Microbial Community as Biological Toolbox for Degradation of Synthetic Polyesters.” Frontiers in Bioengineering and Biotechnology 9: 684459. 10.3389/fbioe.2021.684459.

[asj70063-bib-0031] Ramachandraiah, K. , K. Ameer , G. Jiang , and G. P. Hong . 2022. “Micro‐and Nanoplastic Contamination in Livestock Production: Entry Pathways, Potential Effects and Analytical Challenges.” Science of the Total Environment 844: 157234. 10.1016/j.scitotenv.2022.157234. 35810901

[asj70063-bib-0032] Sandil, S. 2024. Occurrence, Behavior, and Fate of Microplastics in Agricultural and Livestock Wastes and Their Impact on Farmers Fields. Occurrence and Behavior of Emerging Contaminants in Organic Wastes and Their Control Strategies. Elsevier. 10.1016/B978-0-443-13585-9.00001-X.

[asj70063-bib-0033] Selim, N. A. , A. M. El Abd Tawab , A. M. Kholif , H. M. Elsayed , N. E. El‐Bordeny , and E. S. Farahat . 2021. “Impact of the Essential Oils of Marjoram or Basil Dietary Supplementation on Degradability, Ruminal Fermentation and Total Gas Production *In Vitro* .” Egyptian Journal of Nutrition and Feeds 24, no. 1: 85–93. 10.21608/ejnf.2021.170311.

[asj70063-bib-0034] Shelver, W. L. , A. M. McGarvey , and L. O. Billey . 2024. “Disposition of [14C]‐Polystyrene Microplastics After Oral Administration to Lactating Sheep.” Food Additives & Contaminants: Part A 41, no. 9: 1132–1143. 10.1080/19440049.2024.2379382. 39037984

[asj70063-bib-0035] Spanghero, M. , M. Braidot , C. Fabro , and A. Romanzin . 2022. “A meta‐Analysis on the Relationship Between Rumen Fermentation Parameters and protozoa Counts in *In Vitro* Batch Experiments.” Animal Feed Science and Technology 293: 115471. 10.1016/j.anifeedsci.2022.115471.

[asj70063-bib-0036] Su, M. , S. Gan , R. Gao , et al. 2025. “Toxicity Mechanisms of Microplastic and Its Effects on Ruminant Production: A Review.” Biomolecules 15, no. 4: 462. 10.3390/biom15040462. 40305187 PMC12024882

[asj70063-bib-0037] Sun, Y. , X. Ren , J. Pan , et al. 2020. “Effect of Microplastics on Greenhouse Gas and Ammonia Emissions During Aerobic Composting.” Science of the Total Environment 737: 139856. 10.1016/j.scitotenv.2020.139856. 32563113

[asj70063-bib-0038] Tassone, S. , S. Barbera , H. Kaihara , S. Glorio Patrucco , and K. Abid . 2024. “First Evidence of the Effects of Polyethylene Terephthalate Microplastics on Ruminal Degradability and Gastro‐Intestinal Digestibility of Mixed hay.” Animals 14, no. 15: 2139. 10.3390/ani14152139. 39123665 PMC11311064

[asj70063-bib-0039] Tassone, S. , H. Kaihara , S. Barbera , S. Glorio Patrucco , R. Issaoui , and K. Abid . 2025. “Low‐Density Polyethylene Microplastics in the Rumen: Implications for Rumen Fermentation Dynamics and Utilization of Concentrate Feed.” Animals 15, no. 3: 297. 10.3390/ani15030297. 39943067 PMC11815983

[asj70063-bib-0040] Van Soest, P. V. , J. B. Robertson , and B. A. Lewis . 1991. “Methods for Dietary Fiber, Neutral Detergent Fiber, and Nonstarch Polysaccharides in Relation to Animal Nutrition.” Journal of Dairy Science 74, no. 10: 3583–3597. 10.3168/jds.S0022-0302(91)78551-2. 1660498

[asj70063-bib-0041] Wang, Z. , S. Liu , Z. Cheng , et al. 2024. “Endoplasmic Reticulum Stress Exacerbates Microplastics‐Induced Toxicity in Animal Cells.” Food Research International 175: 113818. 10.1016/j.foodres.2023.113818. 38129015

[asj70063-bib-0042] Zhang, Y. , J. Wang , X. Geng , and Y. Jiang . 2021. “Does Microplastic Ingestion Dramatically Decrease the Biomass of Protozoa Grazers? A Case Study on the Marine Ciliate *Uronema marinum* .” Chemosphere 267: 129308. 10.1016/j.chemosphere.2020.129308. 33352364

